# 2-Hy­droxy-4-(prop-2-yn­yloxy)benz­alde­hyde

**DOI:** 10.1107/S1600536812049598

**Published:** 2012-12-08

**Authors:** V. Selvarani, M.A. Neelakantan, V. Silambarasan, D. Velmurugan

**Affiliations:** a Chemistry Research Centre, National Engineering College, K.R. Nagar, Kovilpatti 628 503, India; b CAS in Crystallography and Biophysics, University of Madras, Guindy Campus, Chennai-25, India

## Abstract

The asymmetric unit of the title compound, C_10_H_8_O_3_, contains two independent mol­ecules, both of which are almost planar (r.m.s deviations for all non-H atoms of 0.044 and 0.053 Å). The dihedral angles between the benzene ring and the prop-1-yne group are 3.47 (1) and 3.07 (1)° in the two mol­ecules, and the prop-1-yne groups adopt extended conformations. In each mol­ecule, an intra­molecular O—H⋯O hydrogen bond involving the OH and aldehyde substituents forms an *S*(6) ring. In the crystal, mol­ecules are linked into cyclic centrosymmetric dimers *via* C—H⋯O hydrogen bonds, generating *R*_2_^2^(14) ring motifs. The crystal structure is further stabilized by aromatic π–π stacking inter­actions between the benzene rings [centroid–centroid distances = 3.813 (2) and 3.843 (2) Å]

## Related literature

For the biological activity of benzaldehyde derivatives, see: Zhao *et al.* (2007 ▶); Ley & Bertram (2001 ▶); Delogu *et al.* (2010 ▶). For a related structure see: Esakkiammal *et al.* (2012 ▶). For standard bond lengths, see: Allen *et al.* (1987 ▶) and for hydrogen-bond motifs, see: Bernstein *et al.* (1995) ▶.
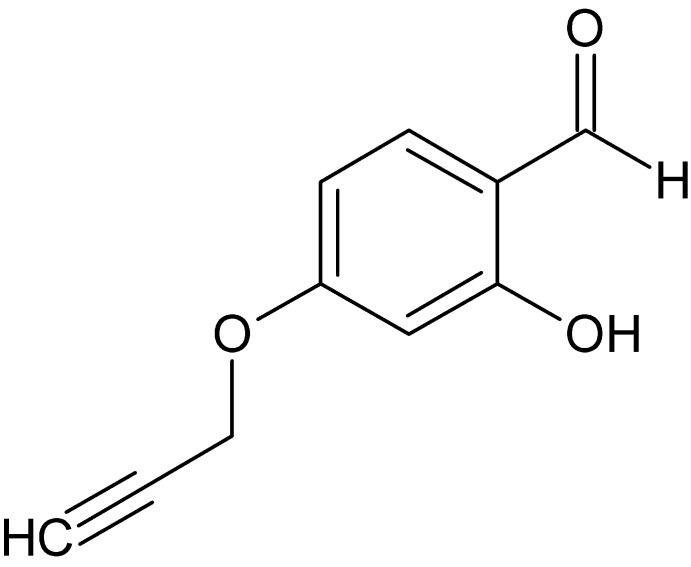


## Experimental

### 

#### Crystal data

C_10_H_8_O_3_
*M* *_r_* = 176.16
Triclinic, 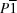

*a* = 7.0835 (5) Å
*b* = 10.4059 (7) Å
*c* = 12.8461 (8) Å
α = 73.910 (3)°
β = 89.756 (4)°
γ = 73.436 (4)°
*V* = 869.16 (10) Å^3^
*Z* = 4
Mo *K*α radiation
μ = 0.10 mm^−1^
*T* = 293 K
0.20 × 0.20 × 0.20 mm

#### Data collection

Bruker SMART APEXII area-detector diffractometer
15699 measured reflections
4347 independent reflections
2880 reflections with *I* > 2σ(*I*)
*R*_int_ = 0.029

#### Refinement

*R*[*F* ^2^ > 2σ(*F* ^2^)] = 0.044
*wR*(*F* ^2^) = 0.132
*S* = 1.05
4347 reflections
243 parameters
H atoms treated by a mixture of independent and constrained refinement
Δρ_max_ = 0.15 e Å^−3^
Δρ_min_ = −0.20 e Å^−3^

### 

Data collection: *APEX2* (Bruker, 2008 ▶); cell refinement: *SAINT* (Bruker, 2008 ▶); data reduction: *SAINT*; program(s) used to solve structure: *SHELXS97* (Sheldrick, 2008 ▶); program(s) used to refine structure: *SHELXL97* (Sheldrick, 2008 ▶); molecular graphics: *ORTEP-3* (Farrugia, 2012) ▶; software used to prepare material for publication: *SHELXL97* and *PLATON* (Spek, 2009 ▶).

## Supplementary Material

Crystal structure: contains datablock(s) global, I. DOI: 10.1107/S1600536812049598/sj5285sup1.cif

Structure factors: contains datablock(s) I. DOI: 10.1107/S1600536812049598/sj5285Isup2.hkl

Supplementary material file. DOI: 10.1107/S1600536812049598/sj5285Isup3.cml

Additional supplementary materials:  crystallographic information; 3D view; checkCIF report

## Figures and Tables

**Table 1:** Hydrogen-bond geometry (Å, °)

*D*—H⋯*A*	*D*—H	H⋯*A*	*D*⋯*A*	*D*—H⋯*A*
O2—H2⋯O3	0.82	1.92	2.6387 (16)	146
O5—H5⋯O6	0.82	1.93	2.6441 (16)	146
C10—H10⋯O3^i^	0.86 (2)	2.51 (2)	3.369 (2)	171.4 (2)
C18—H18*A*⋯O5^ii^	0.97	2.45	3.281 (2)	144
C20—H20⋯O6^i^	0.91 (2)	2.37 (2)	3.280 (2)	178.8 (2)

Symmetry codes: (i) 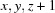; (ii) 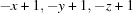.

**Fig. 1.**
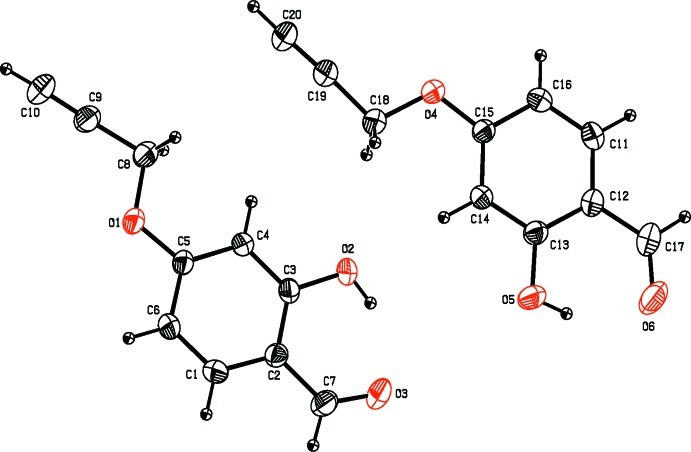
The molecular structure of the title compound, showing the atomic numbering and displacement ellipsoids drawn at the 30% probability level.

**Fig. 2.**
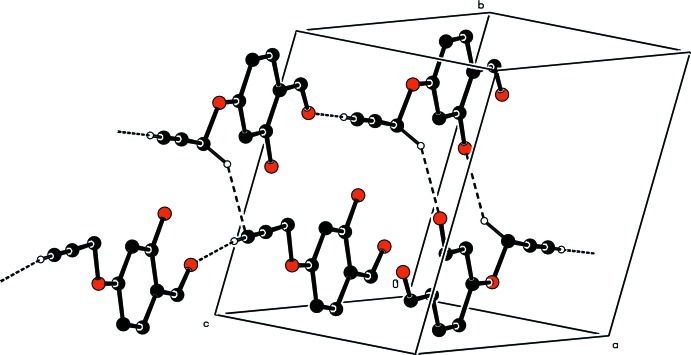
The crystal packing of the molecules viewed down b axis. H atoms not involved in hydrogen bonding (dashed lines) have been omitted for clarity.

## supplementary crystallographic information

### Comment

Schiff bases derived from amines and substituted benzaldehydes exhibit antibacterial, anticancer and antitumour activities (Zhao *et al.*, 2007). Several benzaldoximes, benzaldehyde-*O*-ethyloximes and acetophenone oximes were synthesized and evaluated as tyrosinase inhibitors (Ley & Bertram, 2001). Bis-salicylaldehydes exhibited greater inhibitory activity than salicylaldehyde (Delogu *et al.*, 2010). In view of these potential applications and in continuation of our work on the crystal structures of benzaldehyde derivatives, the structure of the title compound has been carried out and the results are presented here.

X-ray analysis confirms the molecular structure and atom connectivity as illustrated in Fig. 1. The bond distances are normal (Allen *et al.*, 1987) and are comparable with those found in related structures (Esakkiammal *et al.*, 2012). The asymmetric unit contains two crystallographically independent molecules. In both molecules, the dihedral angles between the benzene rings and the prop-1-yne groups are 3.47 (1)° and 3.07 (1)°, respectively. Both molecules are almost planar, with r.m.s deviations of 0.044 and 0.053 Å from the best fit plane through all non-hydrogen atoms in the molecules. The prop-1-yne groups adopt extended conformations as can be seen from the torsion angles C15—O4—C18—C19 = -177.04 (12)° and C5—O1—C8—C9 = 179.39 (13)°. Atoms O2 and O5 deviate by 0.013 (1) and -0.004 (1) Å from the least squares plane of the benzene rings. Intramolecular O2–H2···O3 and O5–H5···O6 hydrogen bonds form S(6) rings in both molecules (Bernstein *et al.*, 1995).

Molecules are linked into cyclic centrosymmetric dimers *via* C–H···O hydrogen bonds with *R*_2_^2^(14) ring motifs (Bernstein *et al.*, 1995). The crystal structure is further stabilized by an aromatic π–π stacking interaction between the benzene rings of adjacent molecules, with centroid to centroid distances *Cg*1···*Cg*2^i^ = 3.813 (2) Å; *Cg*2···*Cg*1^ii^ = 3.843 (2) Å [(i)1 - *x*, 1 - *y*,1 - *z* and (ii) 2 - *x*,1 - *y*,1 - *z*]. (*Cg*1 and *Cg*2 are the centroids of the C1—C6 and C11—C16 rings respectively).

### Experimental

Equimolar amounts of 3-bromopropyne (10 mmol), 2,4-dihydroxybenzaldehyde (10 mmol) and potassium carbonate (15 mmol) were suspended in acetonitrile (30 ml) and refluxed for 30 h in presence of KI (0.1 g) as a catalyst. The reaction mixture was filtered while hot to remove insoluble impurities, neutralized with dil.HCl (3 M), extracted with chloroform and dried with Na_2_SO_4_. The extracts were concentrated to obtain a brown solid which was then purified by column chromatography over SiO_2_ by eluting with a mixture of 4% ethyl acetate in n-hexane. Evaporation of the purified extract yielded 2-hydroxy-4-(prop-2-ynyloxy)benzaldehyde in the form of a pure white solid in 85% yield. Crystals suitable for X-ray analysis were obtined by the slow evaporation method.

### Refinement

The acetylenic H atoms H10 and H20 were located in a difference Fourier map and refined freely. Other H atoms were positioned geometrically (C–H = 0.93–0.97 Å; O–H = 0.82 Å) and allowed to ride on their parent atoms, with *U*_iso_(H) = 1.5*U*_eq_(O) for hydroxyl H atoms and 1.2*U*_eq_(C) for all other H atoms.

### Crystal data

**Table:**

C_10_H_8_O_3_	*Z* = 4
*M**_r_* = 176.16	*F*(000) = 368
Triclinic, *P*1	*D*_x_ = 1.346 Mg m^−^^3^
Hall symbol: -P 1	Mo *K*α radiation, λ = 0.71073 Å
*a* = 7.0835 (5) Å	Cell parameters from 4347 reflections
*b* = 10.4059 (7) Å	θ = 1.7–28.4°
*c* = 12.8461 (8) Å	µ = 0.10 mm^−^^1^
α = 73.910 (3)°	*T* = 293 K
β = 89.756 (4)°	Block, colourless
γ = 73.436 (4)°	0.20 × 0.20 × 0.20 mm
*V* = 869.16 (10) Å^3^	

### Data collection

**Table:**

Bruker SMART APEXII area-detector diffractometer	2880 reflections with *I* > 2σ(*I*)
Radiation source: fine-focus sealed tube	*R*_int_ = 0.029
Graphite monochromator	θ_max_ = 28.4°, θ_min_ = 1.7°
ω and φ scans	*h* = −9→9
15699 measured reflections	*k* = −13→13
4347 independent reflections	*l* = −17→17

### Refinement

**Table:**

Refinement on *F*^2^	Primary atom site location: structure-invariant direct methods
Least-squares matrix: full	Secondary atom site location: difference Fourier map
*R*[*F*^2^ > 2σ(*F*^2^)] = 0.044	Hydrogen site location: inferred from neighbouring sites
*wR*(*F*^2^) = 0.132	H atoms treated by a mixture of independent and constrained refinement
*S* = 1.05	*w* = 1/[σ^2^(*F*_o_^2^) + (0.0595*P*)^2^ + 0.0997*P*] where *P* = (*F*_o_^2^ + 2*F*_c_^2^)/3
4347 reflections	(Δ/σ)_max_ < 0.001
243 parameters	Δρ_max_ = 0.15 e Å^−^^3^
0 restraints	Δρ_min_ = −0.20 e Å^−^^3^

### Special details

**Table:**

Geometry. All e.s.d.'s (except the e.s.d. in the dihedral angle between two l.s. planes) are estimated using the full covariance matrix. The cell e.s.d.'s are taken into account individually in the estimation of e.s.d.'s in distances, angles and torsion angles; correlations between e.s.d.'s in cell parameters are only used when they are defined by crystal symmetry. An approximate (isotropic) treatment of cell e.s.d.'s is used for estimating e.s.d.'s involving l.s. planes.
Refinement. Refinement of *F*^2^ against ALL reflections. The weighted *R*-factor *wR* and goodness of fit *S* are based on *F*^2^, conventional *R*-factors *R* are based on *F*, with *F* set to zero for negative *F*^2^. The threshold expression of *F*^2^ > σ(*F*^2^) is used only for calculating *R*-factors(gt) *etc*. and is not relevant to the choice of reflections for refinement. *R*-factors based on *F*^2^ are statistically about twice as large as those based on *F*, and *R*- factors based on ALL data will be even larger.

### Fractional atomic coordinates and isotropic or equivalent isotropic displacement parameters (Å^2^)

**Table:**

	*x*	*y*	*z*	*U*_iso_*/*U*_eq_	
O4	0.23016 (15)	0.81458 (9)	0.55793 (7)	0.0528 (3)	
C14	0.2775 (2)	0.69318 (13)	0.41998 (10)	0.0473 (3)	
H14	0.3173	0.6058	0.4713	0.057*	
O5	0.31664 (18)	0.58412 (11)	0.28152 (9)	0.0699 (3)	
H5	0.3065	0.6028	0.2151	0.105*	
C16	0.1668 (2)	0.94571 (13)	0.37621 (11)	0.0498 (3)	
H16	0.1326	1.0264	0.3987	0.060*	
C15	0.22699 (19)	0.81361 (13)	0.45238 (10)	0.0423 (3)	
C12	0.2087 (2)	0.83519 (14)	0.23261 (10)	0.0473 (3)	
O6	0.2396 (2)	0.74928 (15)	0.07950 (9)	0.0831 (4)	
C13	0.2683 (2)	0.70387 (14)	0.31045 (11)	0.0467 (3)	
C11	0.1590 (2)	0.95453 (14)	0.26846 (11)	0.0511 (3)	
H11	0.1196	1.0423	0.2175	0.061*	
C18	0.2822 (3)	0.68218 (15)	0.63841 (11)	0.0639 (4)	
H18A	0.4161	0.6290	0.6314	0.077*	
H18B	0.1940	0.6296	0.6284	0.077*	
C19	0.2680 (2)	0.70303 (15)	0.74570 (12)	0.0546 (4)	
C17	0.1995 (2)	0.84683 (19)	0.11911 (12)	0.0643 (4)	
H17	0.1594	0.9367	0.0712	0.077*	
C20	0.2593 (3)	0.71369 (18)	0.83353 (13)	0.0652 (4)	
O1	0.27472 (15)	0.21006 (10)	0.97384 (7)	0.0556 (3)	
C4	0.2102 (2)	0.31928 (13)	0.77968 (10)	0.0480 (3)	
H4	0.1643	0.4086	0.7881	0.058*	
C6	0.3435 (2)	0.06902 (13)	0.85818 (11)	0.0475 (3)	
H6	0.3856	−0.0081	0.9192	0.057*	
C5	0.27430 (19)	0.20366 (13)	0.86926 (10)	0.0426 (3)	
C3	0.2154 (2)	0.30031 (13)	0.67725 (10)	0.0458 (3)	
C2	0.28516 (19)	0.16624 (13)	0.66383 (10)	0.0442 (3)	
C1	0.3487 (2)	0.05198 (14)	0.75674 (11)	0.0480 (3)	
H1	0.3956	−0.0377	0.7491	0.058*	
O2	0.15146 (18)	0.41492 (10)	0.59107 (7)	0.0675 (3)	
H2	0.1609	0.3914	0.5349	0.101*	
O3	0.2389 (2)	0.23901 (13)	0.47217 (8)	0.0756 (4)	
C7	0.2915 (2)	0.14587 (18)	0.55809 (12)	0.0594 (4)	
H7	0.3396	0.0544	0.5542	0.071*	
C9	0.2226 (2)	0.32905 (16)	1.10635 (12)	0.0576 (4)	
C8	0.2136 (3)	0.34536 (16)	0.99018 (12)	0.0664 (4)	
H8A	0.0797	0.3946	0.9582	0.080*	
H8B	0.2996	0.3995	0.9556	0.080*	
C10	0.2270 (3)	0.3192 (2)	1.19909 (14)	0.0684 (5)	
H10	0.230 (3)	0.3082 (19)	1.2682 (16)	0.088 (6)*	
H20	0.253 (2)	0.7250 (17)	0.9014 (15)	0.078 (5)*	

### Atomic displacement parameters (Å^2^)

**Table:**

	*U*^11^	*U*^22^	*U*^33^	*U*^12^	*U*^13^	*U*^23^
O4	0.0719 (7)	0.0437 (5)	0.0408 (5)	−0.0109 (4)	0.0031 (4)	−0.0153 (4)
C14	0.0586 (8)	0.0379 (6)	0.0406 (7)	−0.0080 (6)	0.0023 (6)	−0.0100 (5)
O5	0.0999 (9)	0.0533 (6)	0.0531 (6)	−0.0056 (6)	0.0018 (6)	−0.0275 (5)
C16	0.0560 (8)	0.0390 (7)	0.0518 (8)	−0.0098 (6)	0.0033 (6)	−0.0134 (6)
C15	0.0445 (7)	0.0427 (7)	0.0397 (6)	−0.0110 (5)	0.0038 (5)	−0.0137 (5)
C12	0.0456 (7)	0.0545 (8)	0.0400 (7)	−0.0149 (6)	0.0048 (6)	−0.0106 (6)
O6	0.1047 (10)	0.1030 (10)	0.0461 (6)	−0.0269 (8)	0.0113 (6)	−0.0326 (7)
C13	0.0496 (8)	0.0453 (7)	0.0448 (7)	−0.0095 (6)	0.0041 (6)	−0.0170 (6)
C11	0.0547 (8)	0.0428 (7)	0.0473 (7)	−0.0116 (6)	0.0008 (6)	−0.0025 (6)
C18	0.0978 (12)	0.0474 (8)	0.0417 (7)	−0.0147 (8)	−0.0004 (7)	−0.0118 (6)
C19	0.0653 (9)	0.0533 (8)	0.0459 (8)	−0.0192 (7)	0.0014 (7)	−0.0134 (6)
C17	0.0681 (10)	0.0793 (11)	0.0416 (8)	−0.0221 (8)	0.0062 (7)	−0.0110 (8)
C20	0.0841 (12)	0.0716 (10)	0.0446 (8)	−0.0277 (9)	0.0068 (8)	−0.0195 (8)
O1	0.0775 (7)	0.0464 (5)	0.0335 (5)	−0.0063 (5)	−0.0011 (4)	−0.0091 (4)
C4	0.0655 (9)	0.0367 (6)	0.0382 (7)	−0.0110 (6)	−0.0009 (6)	−0.0093 (5)
C6	0.0544 (8)	0.0390 (6)	0.0422 (7)	−0.0094 (6)	0.0008 (6)	−0.0051 (5)
C5	0.0475 (7)	0.0426 (7)	0.0342 (6)	−0.0106 (5)	0.0010 (5)	−0.0083 (5)
C3	0.0587 (8)	0.0418 (7)	0.0359 (6)	−0.0192 (6)	−0.0013 (6)	−0.0051 (5)
C2	0.0495 (7)	0.0477 (7)	0.0412 (7)	−0.0213 (6)	0.0059 (6)	−0.0150 (5)
C1	0.0528 (8)	0.0392 (7)	0.0524 (8)	−0.0121 (6)	0.0050 (6)	−0.0154 (6)
O2	0.1155 (10)	0.0468 (6)	0.0338 (5)	−0.0239 (6)	−0.0080 (5)	−0.0015 (4)
O3	0.1107 (10)	0.0851 (8)	0.0383 (6)	−0.0384 (7)	0.0061 (6)	−0.0197 (6)
C7	0.0737 (10)	0.0662 (9)	0.0500 (8)	−0.0290 (8)	0.0105 (7)	−0.0264 (7)
C9	0.0645 (10)	0.0590 (9)	0.0495 (8)	−0.0133 (7)	0.0022 (7)	−0.0212 (7)
C8	0.0962 (12)	0.0514 (8)	0.0438 (8)	−0.0067 (8)	0.0006 (8)	−0.0167 (6)
C10	0.0784 (12)	0.0844 (12)	0.0474 (9)	−0.0227 (9)	0.0045 (8)	−0.0286 (8)

### Geometric parameters (Å, º)

**Table:**

O4—C15	1.3591 (14)	O1—C5	1.3634 (14)
O4—C18	1.4237 (16)	O1—C8	1.4245 (16)
C14—C15	1.3798 (17)	C4—C5	1.3797 (17)
C14—C13	1.3806 (18)	C4—C3	1.3825 (18)
C14—H14	0.9300	C4—H4	0.9300
O5—C13	1.3493 (15)	C6—C1	1.3619 (18)
O5—H5	0.8200	C6—C5	1.3935 (17)
C16—C11	1.3619 (18)	C6—H6	0.9300
C16—C15	1.3974 (17)	C3—O2	1.3481 (15)
C16—H16	0.9300	C3—C2	1.3999 (18)
C12—C11	1.3944 (19)	C2—C1	1.3977 (18)
C12—C13	1.4010 (19)	C2—C7	1.4304 (18)
C12—C17	1.4295 (19)	C1—H1	0.9300
O6—C17	1.221 (2)	O2—H2	0.8200
C11—H11	0.9300	O3—C7	1.2251 (19)
C18—C19	1.4522 (19)	C7—H7	0.9300
C18—H18A	0.9700	C9—C10	1.167 (2)
C18—H18B	0.9700	C9—C8	1.454 (2)
C19—C20	1.164 (2)	C8—H8A	0.9700
C17—H17	0.9300	C8—H8B	0.9700
C20—H20	0.911 (18)	C10—H10	0.862 (19)
			
C15—O4—C18	116.91 (10)	C5—O1—C8	117.38 (10)
C15—C14—C13	119.27 (12)	C5—C4—C3	119.00 (12)
C15—C14—H14	120.4	C5—C4—H4	120.5
C13—C14—H14	120.4	C3—C4—H4	120.5
C13—O5—H5	109.5	C1—C6—C5	119.09 (11)
C11—C16—C15	118.97 (12)	C1—C6—H6	120.5
C11—C16—H16	120.5	C5—C6—H6	120.5
C15—C16—H16	120.5	O1—C5—C4	123.96 (11)
O4—C15—C14	123.91 (11)	O1—C5—C6	114.78 (10)
O4—C15—C16	115.03 (11)	C4—C5—C6	121.26 (12)
C14—C15—C16	121.06 (12)	O2—C3—C4	117.85 (12)
C11—C12—C13	118.41 (12)	O2—C3—C2	121.27 (11)
C11—C12—C17	120.70 (13)	C4—C3—C2	120.88 (12)
C13—C12—C17	120.89 (13)	C1—C2—C3	118.26 (11)
O5—C13—C14	117.79 (12)	C1—C2—C7	120.55 (12)
O5—C13—C12	121.53 (12)	C3—C2—C7	121.19 (12)
C14—C13—C12	120.67 (12)	C6—C1—C2	121.50 (12)
C16—C11—C12	121.62 (12)	C6—C1—H1	119.2
C16—C11—H11	119.2	C2—C1—H1	119.2
C12—C11—H11	119.2	C3—O2—H2	109.5
O4—C18—C19	109.46 (11)	O3—C7—C2	125.35 (14)
O4—C18—H18A	109.8	O3—C7—H7	117.3
C19—C18—H18A	109.8	C2—C7—H7	117.3
O4—C18—H18B	109.8	C10—C9—C8	178.40 (17)
C19—C18—H18B	109.8	O1—C8—C9	108.67 (12)
H18A—C18—H18B	108.2	O1—C8—H8A	110.0
C20—C19—C18	177.13 (16)	C9—C8—H8A	110.0
O6—C17—C12	125.79 (15)	O1—C8—H8B	110.0
O6—C17—H17	117.1	C9—C8—H8B	110.0
C12—C17—H17	117.1	H8A—C8—H8B	108.3
C19—C20—H20	178.1 (11)	C9—C10—H10	177.5 (13)
			
C18—O4—C15—C14	−2.4 (2)	C8—O1—C5—C4	−3.0 (2)
C18—O4—C15—C16	177.32 (13)	C8—O1—C5—C6	177.38 (12)
C13—C14—C15—O4	179.71 (12)	C3—C4—C5—O1	−179.63 (12)
C13—C14—C15—C16	0.0 (2)	C3—C4—C5—C6	0.0 (2)
C11—C16—C15—O4	−179.91 (12)	C1—C6—C5—O1	180.00 (12)
C11—C16—C15—C14	−0.2 (2)	C1—C6—C5—C4	0.4 (2)
C15—C14—C13—O5	−179.31 (12)	C5—C4—C3—O2	179.81 (13)
C15—C14—C13—C12	0.0 (2)	C5—C4—C3—C2	−0.3 (2)
C11—C12—C13—O5	179.36 (13)	O2—C3—C2—C1	−179.83 (13)
C17—C12—C13—O5	−0.9 (2)	C4—C3—C2—C1	0.3 (2)
C11—C12—C13—C14	0.0 (2)	O2—C3—C2—C7	0.1 (2)
C17—C12—C13—C14	179.81 (13)	C4—C3—C2—C7	−179.79 (13)
C15—C16—C11—C12	0.3 (2)	C5—C6—C1—C2	−0.4 (2)
C13—C12—C11—C16	−0.2 (2)	C3—C2—C1—C6	0.1 (2)
C17—C12—C11—C16	−179.99 (13)	C7—C2—C1—C6	−179.87 (13)
C15—O4—C18—C19	−177.04 (12)	C1—C2—C7—O3	179.84 (15)
O4—C18—C19—C20	−179 (100)	C3—C2—C7—O3	−0.1 (2)
C11—C12—C17—O6	−179.95 (16)	C5—O1—C8—C9	179.39 (13)
C13—C12—C17—O6	0.3 (3)	C10—C9—C8—O1	−162 (7)

### Hydrogen-bond geometry (Å, º)

**Table:**

*D*—H···*A*	*D*—H	H···*A*	*D*···*A*	*D*—H···*A*
O2—H2···O3	0.82	1.92	2.6387 (16)	146
O5—H5···O6	0.82	1.93	2.6441 (16)	146
C10—H10···O3^i^	0.86 (2)	2.51 (2)	3.369 (2)	171.4 (2)
C18—H18*A*···O5^ii^	0.97	2.45	3.281 (2)	144
C20—H20···O6^i^	0.91 (2)	2.37 (2)	3.280 (2)	178.8 (2)

Symmetry codes: (i) *x*, *y*, *z*+1; (ii) −*x*+1, −*y*+1, −*z*+1.
